# Relationship between lipid layer thickness, incomplete blinking rate and tear film instability in patients with different myopia degrees after small-incision lenticule extraction

**DOI:** 10.1371/journal.pone.0230119

**Published:** 2020-03-12

**Authors:** Yan Li, Shaowei Li, Jihong Zhou, Chang Liu, Man Xu

**Affiliations:** 1 Department of Ophthalmology, Aier School of Ophthalmology, Central South University, Changsha, Hunan Province, China; 2 Department of Ophthalmology, Beijing Aier-Intech Eye Hospital, Beijing, China; National Taiwan University Hospital, TAIWAN

## Abstract

**Objective:**

To elucidate the relationship between lipid layer thickness (LLT), incomplete blinking rate and tear film stability in patients with different myopia degrees after small-incision lenticule extraction (SMILE) and to determine whether there is a difference in the prevalence of dry eye disease (DED) after SMILE among patients with different myopia degrees.

**Methods:**

Fifty patients (100 eyes) were enrolled in this study; they were divided into 3 groups according to the degree of spherical refraction: a low-myopia group (LMG; spherical refraction ≤-3.00 D, 20 eyes), a moderate-myopia group (MMG; -3.00 D < spherical refraction <-6.00 D, 40 eyes), and a high-myopia group (HMG; spherical refraction ≧-6.00 D, 40 eyes). Testing indicators included the ocular surface disease index (OSDI), fluorescein tear film breakup time (FBUT), corneal fluorescence staining (CFS), the Schirmer test (SI), lipid layer thickness (LLT), blink rate (BR) per 20 seconds, incomplete blinking rate, noninvasive keratograph assessment of first and average tear film breakup time (NIKBUT_f_, NIKBUT_av_), and tear meniscus height (TMH). Each indicator was evaluated preoperatively and postoperatively at 1 w, 1 mo and 3 mo.

**Results:**

The mean age was 29.12±5.95 years. There were no significant differences among the three groups (p>0.05), except preoperative age (p = 0.006). There were significant differences in the FBUT among the three groups at postoperative 1 w and 1 mo (p<0.05). There were significant differences in the incomplete blinking rate and FBUT between the LMG and the HMG at postoperative 1 mo (p<0.05). The number of first tear film breakup points located beyond the 6 mm diameter of the cornea was higher in the HMG than in the other groups. The prevalence of DED in the LMG, the MMG, the HMG was 15%, 8% and 23%, respectively, at 1 w postoperative and 30%, 45% and 53%, respectively, at postoperative 1 mo. The change in LLT was significantly correlated with the changes in FBUT (r = 0.408, p<0.001) and incomplete blinking rate (r = -0.266, p = 0.007). The change in OSDI was negatively correlated with the change in SI (r = -0.502, p = 0.000).

**Conclusions:**

The changes in LLT and incomplete blinking rate decreased the stability of the tear film. The changes in LLT, FBUT and incomplete blinking rate differed postoperatively with different myopia degrees. The prevalence of DED was higher in the HMG than in the other two groups.

## Introduction

Dry eye disease (DED) presents a multifactorial pathology at the ocular surface, including tear film changes with or without corneal damage, ocular symptoms, visual degradation, and increased tear osmolarity, which together lead to a reduction in quality of life[1]. DED is the most common complication of corneal refractive surgery[2, 3] and is the main cause of refractive regression, decline in vision quality, and decreased patient satisfaction[4–6].

The lipid layer is located in the outermost layer of the tear film, which helps the tear film respread after blinking and prevents water evaporation. The change in its composition, distribution, and thickness are associated with DED[7]. Blinking plays an important role in maintaining ocular surface integrity, ocular surface moisture, secretion of the lipid layer, and spreading of tear lipids[8–10]. Studies have shown that an increase in incomplete blinking leads to an inadequate lipid distribution and consequent exposure over the inferior ocular surface, which may increase evaporation[11–13].

Small-incision lenticule extraction (SMILE) is a new technique that causes minimal damage to the cornea and corneal nerves and ensures maximal maintenance of anatomical structures and biomechanical properties[14]. SMILE causes little damage to corneal nerve fiber bundles in the treatment area[15]. However, some patients still have symptoms of DED after surgery. The purpose of this study was to elucidate the relationship between lipid layer thickness (LLT), incomplete blinking rate and tear film stability in patients with different myopia degrees after small-incision lenticule extraction (SMILE) and to determine whether there is a difference in the prevalence of dry eye disease (DED) after SMILE among patients with different myopia degrees.

## Materials and methods

### Patients

This study was conducted from November 2018 to May 2019 at the Beijing Aier-Intech Eye Hospital. The study adhered to the tenets of the Declaration of Helsinki and was approved by the Ethics Committee of the Beijing Aier-Intech Eye Hospital (Beijing, China, BJAEYZ201607A01). All participants were fully aware of the purpose, procedures and potential risks associated with the study and signed an informed consent form.

Fifty patients (100 eyes) that underwent SMILE were consecutively included in the study. They were divided into 3 groups according to the degree of spherical refraction: the low-myopia group (spherical refraction ≤-3.00 D, 20eyes), moderate-myopia group (-3.00 D <spherical refraction <-6.00 D, 40 eyes), or high-myopia group (spherical refraction ≧-6.00 D, 40 eyes). The operation was performed by the same individual. The inclusion criteria[16] were as follows: patients who were18- 40 years old, patients with a normal ophthalmic examination; patients who stopped using soft corneal contact lens for more than 2 weeks; patients with a corneal diameter > 6.5 mm; and patients who could be followed. The exclusion criteria were as follows: patients who used any ocular surface drugs on the day of examination; patients who suffered from external ophthalmic diseases or who had undergone external ophthalmic surgery preoperatively; patients who had presented tear embolism preoperatively; and patients with severe DED and an unstable refractive error, ocular abnormalities or disease, progressive myopia or astigmatism or any systemic disease that could affect wound healing (e.g., diabetes). The diagnosis of DED was made using the standard diagnostic criteria published by the Asian Dry Eye Association in 2017: Dry eye symptoms (OSDI≥13) and an FBUT ≦5 s[17]. An examination was performed 3 days preoperatively and 1 w, 1 mo and 3 mo postoperatively. Patients were required to avoid exposure to any ophthalmic treatment and medication 2 hours before the examination.

### SMILE procedure with refractive lenticule extraction

In the SMILE group, a small tunnel incision (4 mm) was created at a 120° angle, and the refractive lenticule was extracted through the incision using a spatula. For all myopic corrections, the optical zone size was 6.5 mm. The depth of the lenticule’s anterior surface to the corneal anterior surface was 120 μm.

### Subjective dry eye symptoms

The symptoms of each subject were evaluated by the ocular surface disease index (OSDI) questionnaire translated into Chinese[18, 19], with a score of 0–100.

### Lipid layer thickness (LLT) measurement, blink rate (BR) and incomplete blinking rate

The LLT was measured by a Lipiview Interferometer (TearScience Inc., Morrisville, NC). The LLT-ave and the BR and incomplete blinking rate in 20 seconds were recorded. Credibility (conformance factor, CF) > 0.80 was required to ensure the accuracy of the data.

### Keratography

The first/average tear film breakup time (NIKBUT_f,_ NITBUT_av_) and tear meniscus height (TMH) were recorded by an Oculus Keratograph 5M instrument. The examination was carried out and repeated 3 times under the condition of natural eye opening, and the average value was recorded.

### Fluorescein tear film break-up time (FBUT) and corneal fluorescence staining (CFS) scoring

Strips of fluorescein sodium (containing 1.0 mg fluorescein sodium) (Jing Ming New Technological Development Co., Ltd., Tianjin, China) were used. To perform the FBUT procedure, patients were told to look up while the tip of the strip quickly touched the inferior conjunctiva. FBUT was calculated by calculating the average of three consecutive breakup times, as determined manually by a stopwatch. After the application of fluorescein, corneal and conjunctival staining was evaluated through a yellow filter using the Oxford scale[20].

### Schirmer secretion testing (SI)

Schirmer tear test strips (Jing Ming New Technological Development Co., Ltd., Tianjin, China) were placed on the outer one-third of the lower temporal conjunctival fornix without anesthesia, and the patient was asked to look down or close their eyelid for 5 minutes. The filter paper strip was removed, and the length of tear immersion was recorded.

### Statistical analysis

Statistical analysis was performed using SPSS 23.0 for Microsoft Windows (Chicago, Illinois, USA). The results were expressed as the mean ± standard deviation (SD). The Kruskal-Wallis test (K-W test) was used to test for abnormally distributed values and Post hoc analysis. Bonferroni corrected P value after multiple comparisons. The correlations were determined by the Spearman correlation test. p < 0.05 was considered to be statistically significant.

## Results

### Basic data analysis

The mean age was 29.12±5.95 years. There were no significant differences among the three groups, except in age (p = 0.006) at preoperative (p > 0.05) (Table 1).

**Table 1:** General information of the study population.

	LMG (N = 20)	MMG (N = 40)	HMG (N = 40)	P
Age(y)	32.30±6.19	27.88±5.09§	28.78±6.20	0.006
Gender (female, (n%))	15(75)	24(60)	29(72.5)	0.368
NCT (mmHg)	16.40±1.57	16.35±1.96	15.30±2.36	0.051
LLT (nanometer)	62.15± 20.83	69.05± 23.09	72.50± 20.54	0.117
BR (times/20S)	7.30±3.37	7.20±2.77	7.30±3.07	0.921
Incomplete blinking rate	0.28±0.27	0.33±0.34	0.28±0.35	0.641
TMH (mm)	0.24± 0.06	0.23± 0.06	0.23± 0.05	0.544
NIKBUT_f_ (seconds)	8.22±3.81	8.22±3.39	8.62±4.77	0.956
NIKBUTav (seconds)	11.68±4.58	11.42±5.71	11.20±4.74	0.897
CFS	0	0	0	1
SI (mm)	11.20±8.24	11.10±6.29	10.33±7.78	0.593
OSDI	6.25±2.17	5.73±2.00	6.20±2.15	0.641
FBUT(s)	6.55± 2.37	6.48± 2.32	6.33± 1.96	0.898

LMG means Low-myopia group; MMG means Moderate-myopia group; HMG means High-myopia group

Data are presented as mean ± SD

Kruskal- Wallis test (K-W test) were used for inter-group test and Post hoc analysis; P < 0.05 was considered statistically significant.

### Pre- and postoperative detection indexes

LLT, BR and FBUT were significantly lower at 1 w and 1 mo postoperatively (p<0.05) than preoperatively. The OSDI values and incomplete blinking rate were significantly higher at 1 w and 1 mo postoperatively than preoperatively (p<0.05) (Table 2).

**Table 2:** Analysis of the pre- and postoperative detection indexes.

	Preoperative	Postoperative 1w 2	Postoperative 1mo 3	Postoperative 3mo 4	P
NCT (mmHg)	15.94±2.11	10.35±1.78*	9.91±1.64#	10.02±1.43‖ǂ†	<0.001
LLT (nanometer)	70.05±20.73	34.71±10.16*	44.42±10.79#§	68.09±21.00ǂ†	<0.001
BR (times/20S)	7.30±2.91	4.20± 2.01*	5.43± 2.11#§	7.04± 2.53ǂ†	<0.001
Incomplete blinking rate	0.30±0.33	0.91± 0.19*	0.57± 0.27#§	0.21± 0.25ǂ†	<0.001
TMH (mm)	0.24±0.05	0.18± 0.04*	0.21± 0.04#§	0.24± 0.05ǂ†	<0.001
NIKBUT_f_ (seconds)	8.43±3.97	3.67± 1.35*	5.27± 2.48#§	10.98±8.89ǂ†	<0.001
NIKBUTav (seconds)	11.48±4.95	6.73± 3.28*	8.74± 3.86#§	13.02± 5.49ǂ†	<0.001
CFS	0	0.02± 0.14	0.01± 0.10	0.02± 0.14	0.528
SI (mm)	10.81± 7.25	---	6.97± 4.89#	9.46± 6.42†	<0.001
OSDI	6.02± 2.09	8.99± 2.55*	11.87± 2.27#§	6.96± 2.10‖ǂ†	<0.001
FBUT (seconds)	6.43± 2.18	3.60± 1.85*	3.89± 1.50#§	6.34± 2.52ǂ†	<0.001

Kruskal- Wallis test (K-W test) was used for inter-group test and Post hoc analysis; P < 0.05 was considered statistically significant.

Bonferroni corrected P value after multiple comparisons; P < 0.0083 was considered statistically significant.

*Indicates a statistically significant difference between groups 1 and 2 with P <0.0083

#Indicates a statistically significant difference between groups 1 and 3with P <0.0083.

‖Indicates a statistically significant difference between groups 1 and 4 with P <0.0083.

§Indicates a statistically significant difference between groups 2 and 3with P <0.0083.

ǂ Indicates a statistically significant difference between groups 2 and 4 with P <0.0083.

†Indicates a statistically significant difference between groups 3 and 4 with P <0.0083.

### Changes in various pre- and postoperative indexes in patients with different degrees of myopia

There was significant difference in FBUT among the three groups at 1 w and 1 mo postoperatively (p<0.05). There were significant differences in FBUT and incomplete blinking rate between the LMG and the HMG at 1 mo postoperatively (p<0.05). Although LLT was higher in the HMG at 1 mo postoperatively than in the other two groups, and there were differences in LLT among the three groups (p = 0.043), no significant difference was found between any of the two groups after adjusting the P value (p > 0.05) (Table 3).

**Table 3:** Analysis of the detection indexes in the different groups postoperatively.

	Postoperative 1w				Postoperative 1mo				Postoperative 3mo			
	LMG	MMG	HMG	p	LMG	MMG	HMG	p	LMG	MMG	HMG	p
NCT (mmHg)	10.70±1.81	16.35±1.96	10.20±1.98	0.770	10.15±2.03	10.00±1.32	9.90±1.67	0.667	10.20±1.44	10.30±1.18	9.59±1.60	0.140
LLT((nanometer))	31.90± 7.43	33.00± 9.24	37.83± 11.52	0.056	40.80±10.16	43.05± 10.64	47.68± 11.09	0.043	59.30±16.14	69.5± 20.35	69.08± 21.13	0.157
BR (times/20S)	4.35±1.96	4.40±1.91	3.93±2.15	0.314	5.05±1.00	5.50±2.20	4.98±2.40	0.190	7.95±2.95	7.38±2.37	6.25±2.28*	0.029
Incomplete blinking rate	0.94±0.10	0.90±0.23	0.90±0.17	0.930	0.68±0.17	0.59±0.29	0.50±0.27§	0.042	0.30±0.27	0.19±0.23	0.18±0.24	0.051
TMH (mm)	0.18± 0.04	0.18± 0.04	0.18± 0.04	0.981	0.21± 0.05	0.21± 0.04	0.21± 0.03	0.938	0.26± 0.06	0.23± 0.05	0.24± 0.04	0.130
NIKBUT_f_(s)	3.93±1.41	3.92±1.47	3.30±1.12	0.059	5.62±2.11	5.48±1.77	4.34±1.49§	0.009	9.63±5.73	9.51±3.91	9.46±5.36	0.652
NIKBUTav (s)	7.21±3.68	7.03±3.71	6.18±2.53	0.638	8.37±3.49	8.41±2.73	8.52±3.96	0.951	12.18±5.87	12.80±4.66	12.55±5.26	0.602
CFS	0	0.03±0.16	0.03±0.16	0.777	0	0.03±0.16	0	0.472	0.05±0.22	0	0.03±0.16	0.413
SI (mm)	---	---	---	---	6.45±4.85	7.50±5.42	6.70±4.40	0.655	9.75±7.63	9.63±6.19	9.15±6.15	0.917
OSDI	8.45±2.76	9.00±2.23	9.25±2.76	0.325	11.10±2.40	11.58±1.60	11.78±2.63	0.431	6.35±2.58	6.73±1.74	7.50±2.10	0.153
FBUT (seconds)	6.25± 1.29	3.33± 1.65*	2.55± 1.60§	<0.001	5.00± 1.12	3.80± 1.11*	3.43± 1.72§	<0.001	6.50± 2.28	6.35± 1.69	6.20± 2.72	0.647

LMG means Low-myopia group; MMG means Moderate-myopia group; HMG means High-myopia group

s means seconds

Kruskal- Wallis test (K-W test) were used for inter-group test and Post hoc analysis; P < 0.05 was considered statistically significant.

Bonferroni corrected P value after multiple comparisons; P < 0.017 was considered statistically significant.

*Indicates a statistically significant difference between groups LMG and MMG with P <0.017.

#Indicates a statistically significant difference between groups MMG and HMG with P <0.017.

§Indicates a statistically significant difference between groups LMG and HMG with P <0.017.

### Prevalence of DED and changes in the location of the first tear film breakup point pre- and postoperatively in patients with different myopia degrees

**Fig 1:**
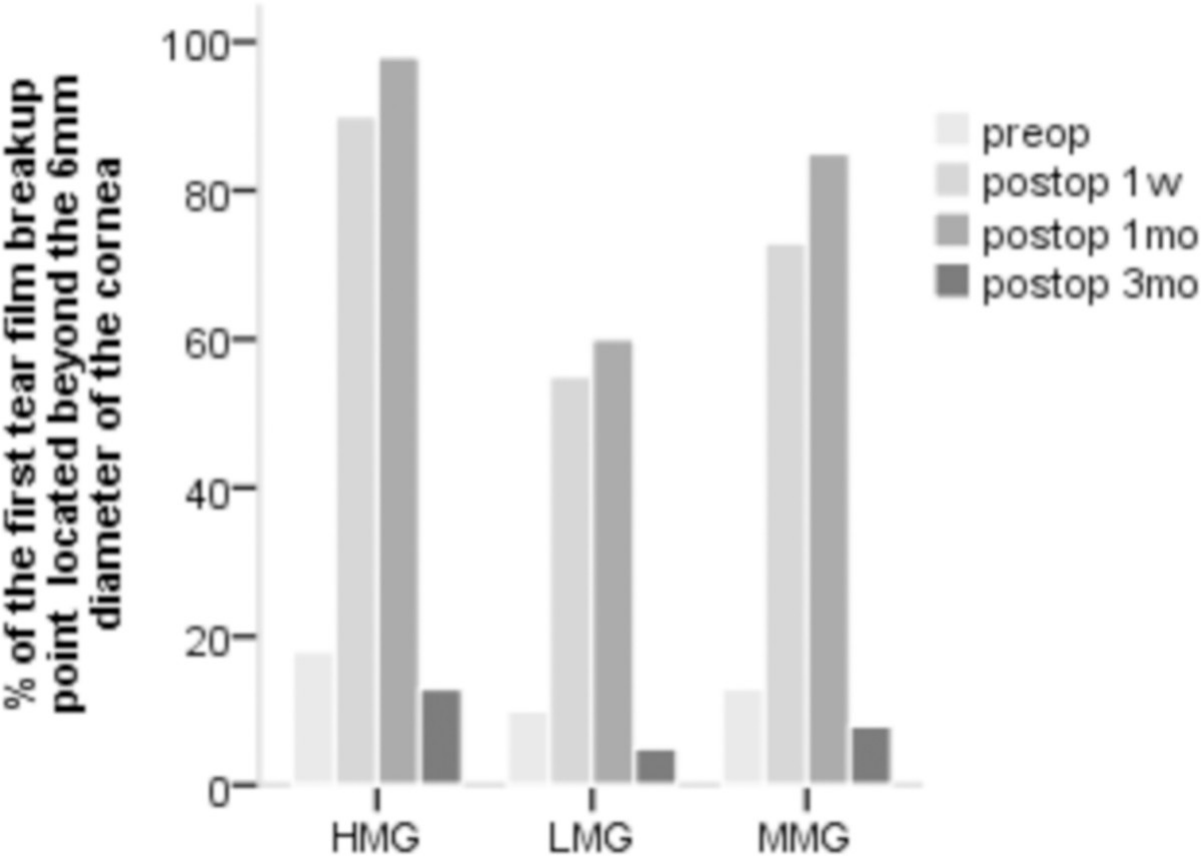
The location of the first tear film break-up point in the three groups.

The prevalence of DED in the LMG, the MMG, the HMG was 15%, 8% and 23%, respectively, at postoperative 1 w and 30%, 45% and 53%, respectively, at postoperative 1 mo. The value of the first tear film breakup point located beyond the 6 mm diameter of the cornea in the HMG was higher than that in the other two groups (Fig 1).

### Correlation analysis

**Fig 2:**
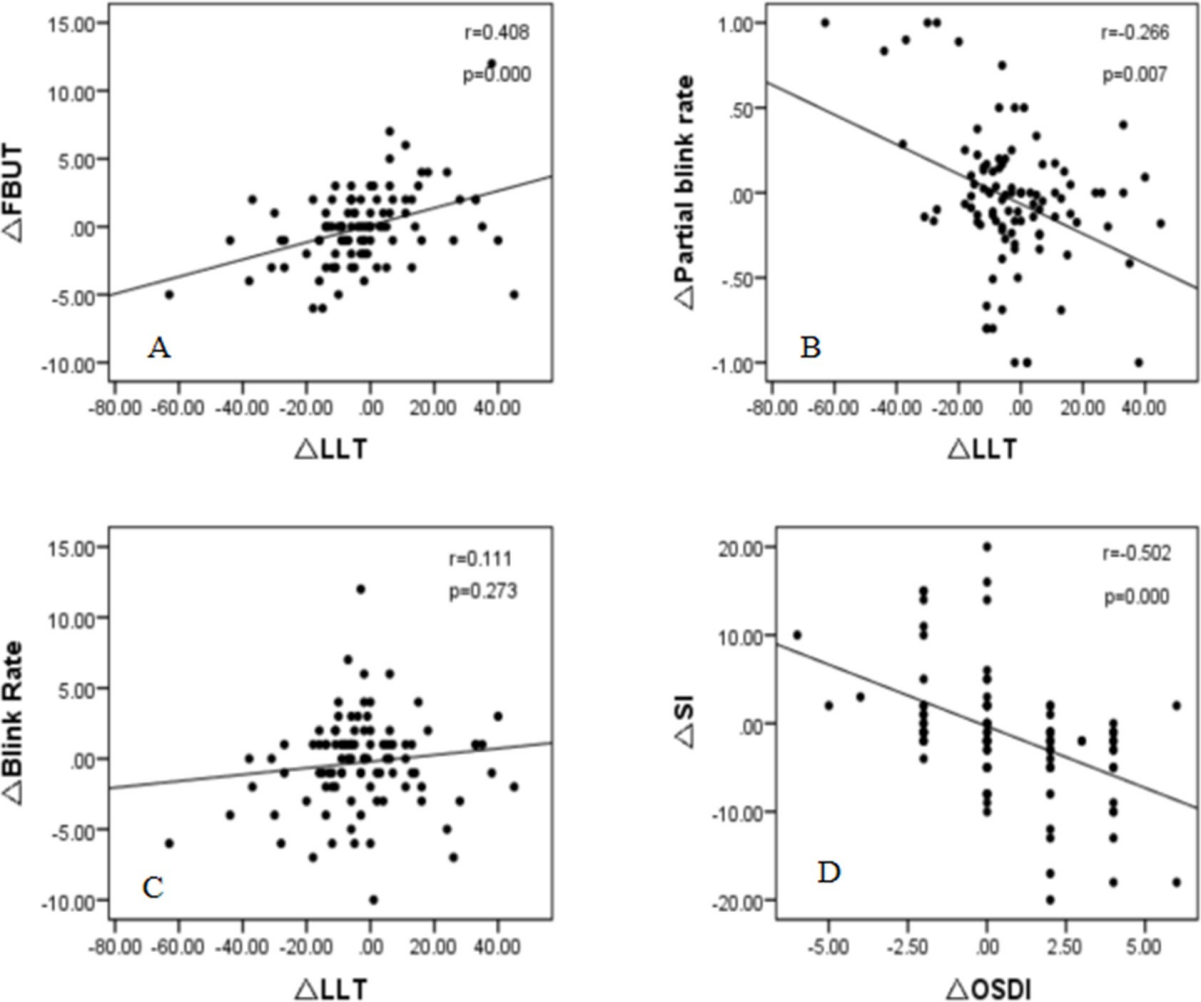
Correlation between the changes in LLT and FBUT (A), the changes in LLT and incomplete blinking rate (B), the changes in LLT and blink rate (C), and the changes in OSDI and SI (D) from preoperative to 3-month postoperative measurements.

In this study, the changes in values between preoperative and 3 mo measurements were represented as delta indexes. Correlation analysis was performed for changes at postoperative 3 mo. The change in LLT was significantly correlated with the changes in FBUT (r = 0.408, p<0.001) and incomplete blinking rate (r = -0.266, p = 0.007). There was no correlation between the change in LLT and the change in blink rate (r = 0.111, p = 0.273). The change in OSDI showed a negative correlation with the change in SI (r = -0.502, p = 0.000). There was no correlation between the changes in LLT and age (r = 0.120, p = 0.233) (Fig 2).

## Discussion

It has been reported that 95% of patients have symptoms of DED immediately after undergoing laser-assisted in situ keratomileusis (LASIK), 60% of patients have DED symptoms 1 mo after LASIK[21], and 10–40% of patients have DED symptoms that can last for 6 mo after LASIK[22–24]. Most research has focused on the prevalence of DED symptoms, although the questionnaire that has been used has differed among studies, leading to slightly different results. Qiu et al.[25] used three symptoms as symptom scoring criteria, and the results showed that 56% of patients had dry eye symptoms at 1 w after SMILE operation. Zhang et al.[26] used eight symptoms in the symptom questionnaire, and the results showed that 37% of patients had dry eye symptoms at 1 mo postoperatively. According to the dry eye diagnosis standard published by the Asian Dry Eye Association in 2017, we found that the prevalence of DED was 15% at 1 w and 45% at 1 mo postoperatively. The recovery time for DED was the same as that reported by Shah et al.[27] and Qiu et al.[25].

In our study, the OSDI represents the subjective dry eye symptom score of the patient. Although the OSDI increased postoperatively, the mean OSDI remained within the normal range (OSDI <13). In addition, there was no significant change in mean OSDI among the three groups, indicating that SMILE surgery caused only slight aggravation of dry eye symptoms.

FBUT is the principal index that reflects the stability of the tear film. At 1 w and 1 mo postoperatively, FBUT was significantly lower than the preoperative value in all three groups, which is consistent with the report of Vestergaard et al.[28]. Consistent with the results of Li et al.[29], FBUT returned to its preoperative value 3 mo after SMILE. However, we found that FBUT in the HMG was significantly lower than that in the LMG and the MMG at 1 w and 1 mo postoperatively, indicating that tear film stability in the HMG was poorer. Studies have shown that abnormal morphology of the anterior surface of the cornea can affect the distribution of the tear film. The higher the corrected diopter, the greater the central cutting depth needs to be; moreover, the change in the corneal aspheric surface is significant, and the rate of irregularities increase after SMILE surgery[30]. Hong et al.[31] reported that the first tear film breakup point (the thinnest part of the tear film) was located at the steepest part of the cornea, i.e., the area with the largest corneal curvature. Bower et al.[32] reported that irregularity in the anterior surface of the cornea was associated with DED. The corneal surface morphology in patients with high myopia markedly changed postoperatively due to the relatively deep cut in the corneal stroma layer. This resulted in increased tear film instability and an increased ratio of the first tear film breakup point, which was located beyond the 6 mm diameter of the cornea in the HMG.

The SI value and TMH reflect the secretory function of the lacrimal glands. SMILE requires a small incision (2.0 mm), which retains the integrity of the superficial corneal nerve to a large extent. The anatomical structure and biological function of the cornea were maintained[29]. SI and TMH were significantly lower at postoperative 1 mo than preoperatively in the three groups, but there was no significant difference among the three groups. Some studies have shown that corneal sensitivity is not significantly changed postoperatively after SMILE surgery[33]. These findings suggested that the effect of the operation on the corneal nerves is small, the production and secretion of the tear is not affected, and the effect of cutting depth on tear secretion function after SMILE surgery is minimal.

Our study showed that the age of patients was significantly higher in the LMG than in the MMG. But there was no significant difference in LLT, PB, FBUT and incomplete blinking rate among different age groups at pre and postoperative (p>0.05). There was no significant difference in gender distribution among the three groups (p>0.05). The results of the study on the effects of age and gender on LLT were not consistent. Maïssa et al.[34] measured the LLT based on its appearance using the Tearscope found that in the 45 years and over age group, age was associated with a significantly thinner LLT. A significantly thinner lipid layer was observed in older women (≥45 years) than in older men (≥45 years). The study of Jung et al.[35] confirmed that in normal subjects, age was significantly associated with LLT, but gender showed no significant impact on LLT; in patients with DES, increased age and female sex were positively associated with LLT. Different results from previous studies may be related to different subjects and different measuring instruments. The study of Schallhorn et al.[36] found that the symptoms of DED had no significant correlation with age. More recent data now suggest that neither gender nor age influences the risk for dry eyes post corneal refractive surgery[37, 38]. The higher average age in the LMG may be related to the tolerance to myopia degree and the choice of operation timing.

The lipid layer is located at the outermost portion of the tear film. Its functions includes refraction, reducing tear evaporation, lubricating the eye surface, blocking pathogenic microorganisms, and preventing tear outflow during sleep[39]. Thus, LLT is an important index for evaluating the stability of the tear film[35]. According to the results of Goto et al.[40], LLT decreased significantly at 1 w after SMILE and returned to the preoperative level after 3 mo. However, Chen et al.[41] found that there was no significant change in LLT, total blink rate and the incomplete blinking rate at 1 mo after LASIK. However, our study found that LLT decreased with increasing incomplete blinking rate after SMILE surgery. The lipid layer is secreted by meibomian glands, and it is difficult to induce changes in meibomian glands within a short period after corneal refractive surgery[41]. In addition, Wang et al.[42] found that poorer lipid layer quality was observed in participants who exhibited partial blinking. Other studies have shown that with the incomplete blinking, the delivery of meibomian lipids to the tear film reduced [8, 10, 43, 44]. Furthermore, blinking facilitates the spreading and distribution of the lipid layer across the ocular surface, and partial blinking can result in poor maintenance of the lipid layer integrity[10, 44]. Together with the increased incomplete blinking rate, the impaired lipid layer quality may potentially trigger a vicious cycle of ocular surface instability, which can further exacerbate the signs and symptoms of DED[42]. Analysis of the detection indexes in different groups after SMILE surgery showed that the incomplete blinking rate was significantly higher in the LMG as compared to the HMG at 1 mo postoperatively. Correlation analysis showed that the change in LLT was negatively correlated with the change in incomplete blinking rate. However, Although LLT was higher in the HMG at 1 mo postoperatively than in the other two groups, there was no significant difference between any of the two groups after adjusting the P value. This may be related to the limited measurement range of LLT and the different sample size of the groups. In addition, LLT in each group was found to be increase with the decrease of incomplete blinking rate. The difference of LLT among the three groups decreases with the time after SMILE surgery, these may be related to the effect of incomplete blinking rate on LLT and the recovery of tear film stability.

Some studies have shown that LLT may be a protective factor of tear film stability after CRS. The correlation analysis showed that the change in LLT was negatively correlated with the change in FBUT. However, we found that FBUT was significantly lower in the HMG than in the other two groups. The average thickness of the lipid layer was measured in our study. A Lipiview Interferometer was used to measure the average lipid layer thickness between the lower margin of the pupil and the lower eyelid margin, is not the average thickness of the whole tear film lipid layer, and the composition and distribution of the lipid layer were not detected. Therefore, although the thickness of the lipid layer has a certain protective effect on the stability of the tear film, it is worth noting that the effect of the lipid layer on the tear film is complex. The quality, quantity, distribution, and composition of the lipid layer are all related to its function[41].

There are some limitations to this study. First, no changes in corneal sensitivity pre- and post-SMILE were measured, and whether there was any difference in corneal sensitivity postoperatively with different myopia degrees has not been studied. Second, the statistical deviation caused by the limited number of low-myopia patients may have affected the accuracy of the results. Third, we only studied the effect of incomplete blinking rate and LLT on tear film stability, the role of tear film composition and distribution after SMILE needs to be further studied.

## Conclusion

In conclusion, the changes in LLT and incomplete blinking rate decreased the stability of the tear film. The changes in LLT, FBUT, incomplete blinking rate and the location of the first tear film breakup point differed postoperatively for different degrees of myopia. The prevalence of DED after SMILE varied among patients with different degrees of myopia. Well directed blink training is beneficial for the secretion of the lipid layer and the recovery of DED.

## Supporting information

S1 Data (XLS)
